# Thymosin Alpha1-Fc Modulates the Immune System and Down-regulates the Progression of Melanoma and Breast Cancer with a Prolonged Half-life

**DOI:** 10.1038/s41598-018-30956-y

**Published:** 2018-08-17

**Authors:** Fanwen Wang, Tingting Yu, Heng Zheng, Xingzhen Lao

**Affiliations:** 1 School of Life Science and Technology, China Pharmaceutical University, Nanjing, 210009 P.R. China; 2 Dongyangguang pharmaceutical r&d co. LTD, Dongguan, 523000 P.R. China

## Abstract

Thymosin alpha 1 (Tα1) is a biological response modifier that has been introduced into markets for treating several diseases. Given the short serum half-life of Tα1 and the rapid development of Fc fusion proteins, we used genetic engineering method to construct the recombinant plasmid to express Tα1-Fc (Fc domain of human IgG4) fusion protein. A single-factor experiment was performed with different inducers of varying concentrations for different times to get the optimal condition of induced expression. Pure proteins higher than 90.3% were obtained by using 5 mM lactose for 4 h with a final production about 160.4 mg/L. The *in vivo* serum half-life of Tα1-Fc is 25 h, almost 13 times longer than Tα1 in mice models. Also, the long-acting protein has a stronger activity in repairing immune injury through increasing number of lymphocytes. Tα1-Fc displayed a more effective antitumor activity in the 4T1 and B16F10 tumor xenograft models by upregulating CD86 expression, secreting IFN-γ and IL-2, and increasing the number of tumor-infiltrating CD4+ T and CD8+ T cells. Our study on the novel modified Tα1 with the Fc segment provides valuable information for the development of new immunotherapy in cancer.

## Introduction

Since the discovery of thymosin alpha 1 (Tα1) in the 1970s, several studies have been investigating on Tα1. Tα1 (brand name: ZADXIN, INN: thymalfasin) is a small molecule polypeptide with 28 amino acids at about 3.1 kDa^1^. Tα1 acts through Toll-like receptors (TLR2 and TLR9) in myeloid and plasmacytoid DCs (dentritic cells)^2^, leading to the activation and differentiation of DCs and T cells, as well as the initiation of cytokines, such as interferon-gamma (IFN-γ) and interleukin-2 (IL-2)^3^. Also, Tα1 can antagonize the dexamethasone (DEX)-induced apoptosis of CD4+CD8+ thymocytes^4^ and the hydrocortisone (HC)-induced decrease in the thymus index and spleen index^5^. Moreover, Tα1 has been evaluated for its activities in hepatitis B and C^6–8^, cystic fibrosis^9^, cancer^10,11^, immune deficiency^12^, and HIV/AIDS^13^. The short serum half-life of Tα1 is no more than 2 h with a poor tumor penetration that limits its clinical use. Combinations of Tα1 and peginterferon α-2a as well as of Tα1 and DEX have made some achievements^14,15^.

Among the strategies of extending serum half-life in the body, adding an immunoglobin G (IgG) Fc fragment is one of the most effective technologies. The Fc fragment exhibits therapeutic improvement by interacting with FcRn resulting in the delayed lysosomal degradation of immunoglobulins by cycling them back into circulation and in a prolonged half-life as described above^16–18^. In the production aspect, recombinant expression of Fc-fusion proteins offer a relatively high content^16^. Moreover, Fc region can be leveraged for its high reversible affinity to staphylococcal protein A or streptococcal protein G^19^. His6-tag was introduced into the fusion protein for purification by using nickel ion affinity chromatography. So far, 11 Fc-fusion proteins have been approved by FDA^20^ and more than 300 have been studied.

In this study, Tα1-Fc is designed by introducing the C-terminus of Tα1 to the hinge of IgG4 Fc for the extension of half-life. The recombinant protein was investigated on an optimum induced condition and further be purified for the next study on *in vivo* activities. Rats were treated by vein injection to determine the half-life. Moreover, anti-tumor activity was evaluated on 4T1 and B16F10 xenograft tumor models by exploring Tα1-Fc effects on tumor inhibition and cytokine expression.

## Results

### The optimum expression condition of Tα1-Fc

**Figure 1:**
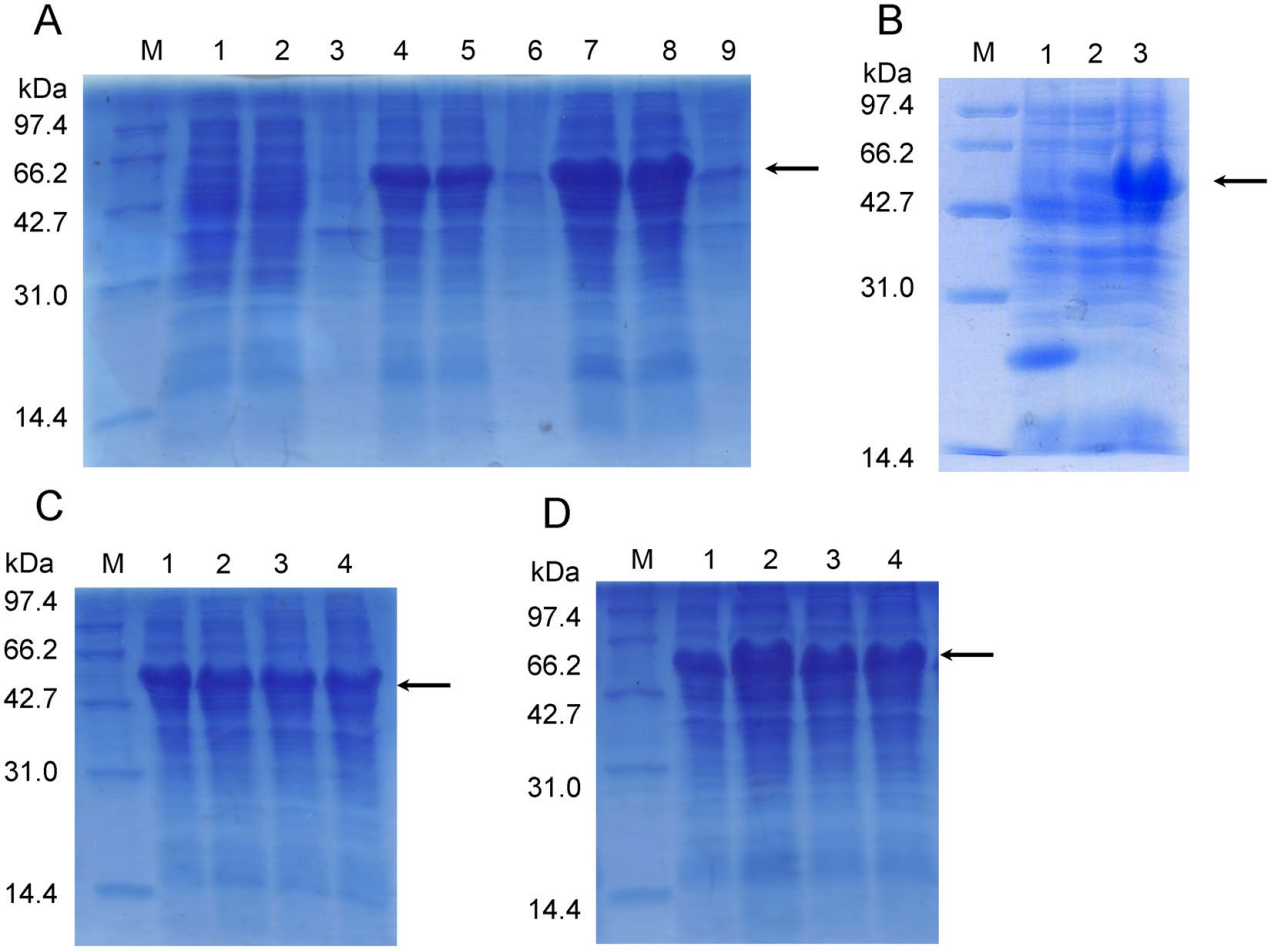
The optimum induced condition of recombinant protein. (**A**) The bacteria solution in the logarithmic growth phase are induced with 1 mM IPTG or 5 mM lactose for 4 h. Lane 1–3: total protein, supernatant, precipitation of none inducer; Lane 4–6: total protein, supernatant, precipitation of 1 mM IPTG; Lane 7–9: total protein, supernatant, precipitation of 5 mM lactose. (**B**) Lane 1–3: total protein the empty pET32a vector, no inducer, 5 mM lactose for 4 h. (**C**) Lane 1–4: 2.5 mM, 5 mM, 7.5 mM, 10 mM lactose for 4 h. (**D**) Lane 1–4: 2 h, 4 h, 6 h, 8 h with 5 mM lactose.

**Figure 2:**
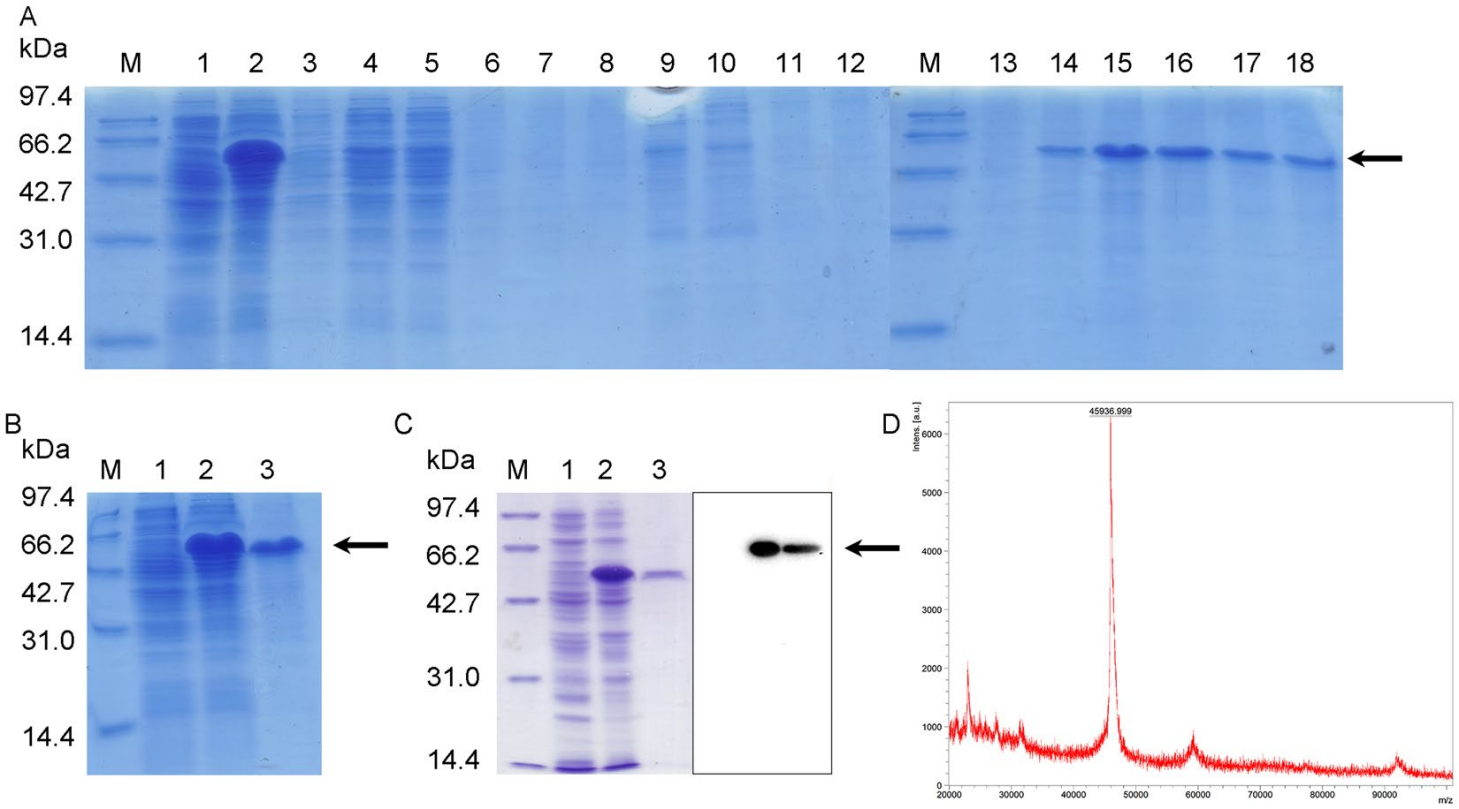
Tα1-Fc was proved to be correct. (**A**) The supernatant of bacteria was purified by subjected to Ni^2+^ affinity chromatography. Lane 1–2: none inducer, 5 mM lactose for 4 h; Lane 3: effluent solution; Lane 4–6: 0 mM imidazole; Lane 7–9: 25 mM imidazole; Lane 10–12: 50 mM imidazole; Lane 13–15: 100 mM imidazole; Lane 16–18: 200 mM imidazole. (**B**) Lane 1–3: none inducer, 5 mM lactose for 4 h, purified solution. (**C**) Lane 1–3: none inducer, 5 mM lactose for 4 h, purified solution. (**D**) Mass spectrometry analysis of Tα1-Fc.

Plasmid pET32a (+) with inserted Trx tag and His_6_ tag was used as a proper expression vector for soluble fusion protein expression^21^. This study, as a single-factor experiment, was performed using IPTG or lactose with different induction times and determined by SDS-PAGE following ImageJ analysis. The fusion protein was expressed in the supernatant by using 1 mM IPTG and 5 mM lactose with a protein content of about 30.5% and 33.3%, respectively, which suggest a soluble expression (Fig. 1A) (Fig. 1A and B gels cropped from different parts of the same gel, full-length of Fig. 1A and B gels corresponding to Supplementary Fig. S1); the molecular weight ranged from 42.7 kDa to 66.2 kDa, which are consistent with the theoretical value. Figure 1B (see Supplementary Fig. S2) was performed to exclude the interference of empty pET32a vector induced expression. Proteins about 17 kDa was mainly expressed in the supernatant of negative control. And there is negligible impact of vector itself on the expression of Tα1-Fc. With an increased lactose concentration (i.e., 2.5 mM, 5 mM, 7.5 mM, and 10 mM), the protein contents were 21.6%, 22.3%, 18.6% and 18.3%, respectively (Fig. 1C) (see Supplementary Fig. S1). With the gradual extension of the induction time (i.e., 2 h, 4 h, 6 h, and 8 h), the protein content was about 23.2%, 37.8%, 30.5%, and 28.8% (Fig. 1D) (see Supplementary Fig. S3); hence, the following induced expression was performed for 4 h. In summary, this soluble expression of recombinant Tα1-Fc in Escherichia coli reached the highest level of about 45.8% when incubated with 5 mM lactose for 4 h (Fig. 2B) (see Supplementary Fig. S5).

### Identification of Tα1-Fc

With the increased concentration of imidazole, the competition of imidazole with the polyhistidine tag in binding with the Ni^2+^ column gets stronger, and the content of the target protein being eluted increased. In Fig. 2A (Fig. 2A gel cropped from different gels, full-length of Fig. 2A see Supplementary Fig. S4 and Supplementary Fig. S5), clear bands can be seen with 100 mM and 200 mM imidazole, especially 200 mM. Therefore, the solution eluted by 100 mM and 200 mM imidazole was collected and then desalted using Sephadex G-25. The lyophilized powder is white floc, and protein production reached 160.4 mg/L. Contents of the induced protein, purified protein, and lyophilized powder were 45.8%, 90.3%, and 92.5%, respectively (Fig. 2B). The expressed product shows an ability to combine with IgG4 (Fig. 2C). Moreover, mass spectrometry analysis result showed that the MW of this fusion protein Tα1-Fc is about 45937 Da which is very close to the theoretical MW value(Fig. 2D). In general, this protein that we induced is the one that we designed.

### Tα1-Fc shows a longer serum half-life

**Figure 3:**
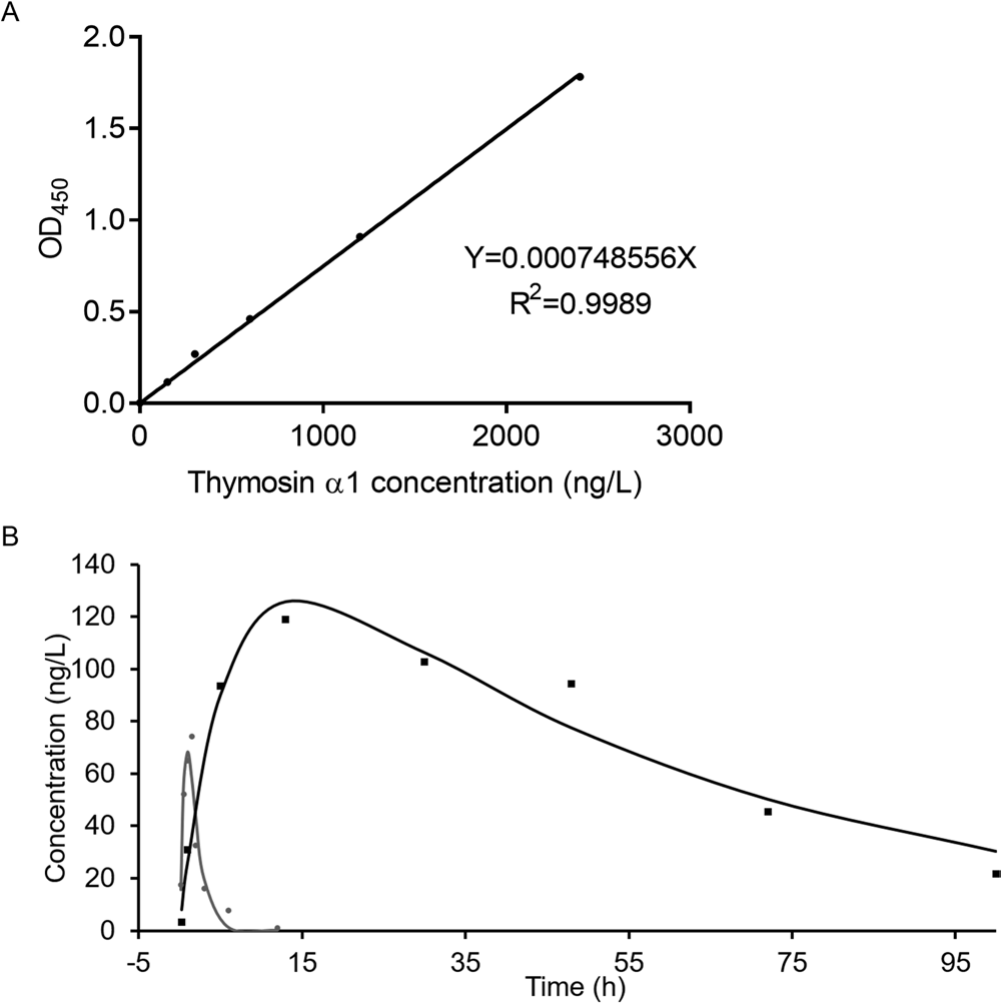
The serum half-life was performed on Wistar rat by injecting to tail vein at a single dose and detected by human Tα1 ELISA kit. (**A**) The standard curve of Tα1. (**B**) The drug concentration and time curve of Tα1 and Tα1-Fc. The half-life of Tα1 and Tα1-Fc are 1.933 h and 24.576 h respectively (n = 3).

Half-life extension is dominated by strategies utilizing albumin binding or fusion; which is the fusion of an immunoglobulin Fc region and PEGylation. Result shows that the serum half-life of Tα1-Fc was 24.58 h, which is almost 13 times longer than of Tα1 (Fig. 3). Peak concentration of Tα1 and Tα1-Fc occurred at 1.5 and 13 h with a concentration of 74.347 ng/L and 118.896 ng/L, respectively. The relative bioavailability of Tα1-Fc is about 90.70% (Table 1). The implementation of half-life extension strategies allows the generation of long-lasting therapeutics with improved pharmacokinetic and pharmacodynamic properties^22^.

**Table 1:** Pharmacokinetic parameters of Tα1-Fc after a single intravenous injection (n = 3).

Two-compartment model	Tα1	Tα1-Fc
Cmax^a^ (ng/L)	74.347	118.896
Tmax^b^ (h)	1.5	13
t1/2z^c^ (h)	1.933	24.576
AUC(0-∞)^d^ (ng/L*h)	191.934	8146.158
V_d_^e^ (L/kg)	0.025	0.011
F^f^ (%)	98.69	90.70

^a^Maximum concentration; ^b^time to maximum concentration; ^c^elimination half-life;

^d^area under concentration-time curve; ^e^apparent volume of distribution; ^f^absolute bioavailability.

### Tα1-Fc restored immune system of immunocompromised mice induced by hydrocortisone

Hydrocortisone is a glucocorticoid that can affect the level of endogenous GCs and interfere with the proliferation and differentiation of lymphocytes resulting in immune injury. As the spleen and the thymus are the main immune organs of the human body, immunocompromised mice model was built by injecting HC for a week and then were treated by drugs to examine the immunological activity of the fusion protein. In Table 2, the spleen index and thymus index decreased sharply when treated with HC. The spleen index of the drug group increased to 7.12–7.69 mg/g, which is close to the blank group compared with the normal control group (PBS). A significant difference was found in the spleen index and the thymus index of Tα1-Fc (p < 0.01). The thymus index of Tα1-Fc was 0.87 ± 0.21 mg/g, whereas 0.60 ± 0.23 mg/g of Tα1 compared with 0.59 ± 0.14 mg/g of PBS. Similarly, the spleen index also abided by the order of Tα1-Fc, Tα1, PBS from strong to weak; these two indexes suggest that Tα1-Fc has a stronger activity in repairing the impaired spleen and impaired thymus than Tα1.

**Table 2:** The spleen index and thymus index in immunocompromised mice model (n = 8).

Treatment	Spleen index (mg/g)	Thymus index (mg/g)
HC + PBS	4.40 ± 1.47	0.56 ± 0.14
HC + Tα1	7.12 ± 2.03**	0.60 ± 0.23
HC + Tα1-Fc	7.69 ± 0.84**	0.87 ± 0.21**
Normal control	8.14 ± 1.71**	1.57 ± 0.45**

The difference was analyzed compared with HC + PBS group.

**Figure 4:**
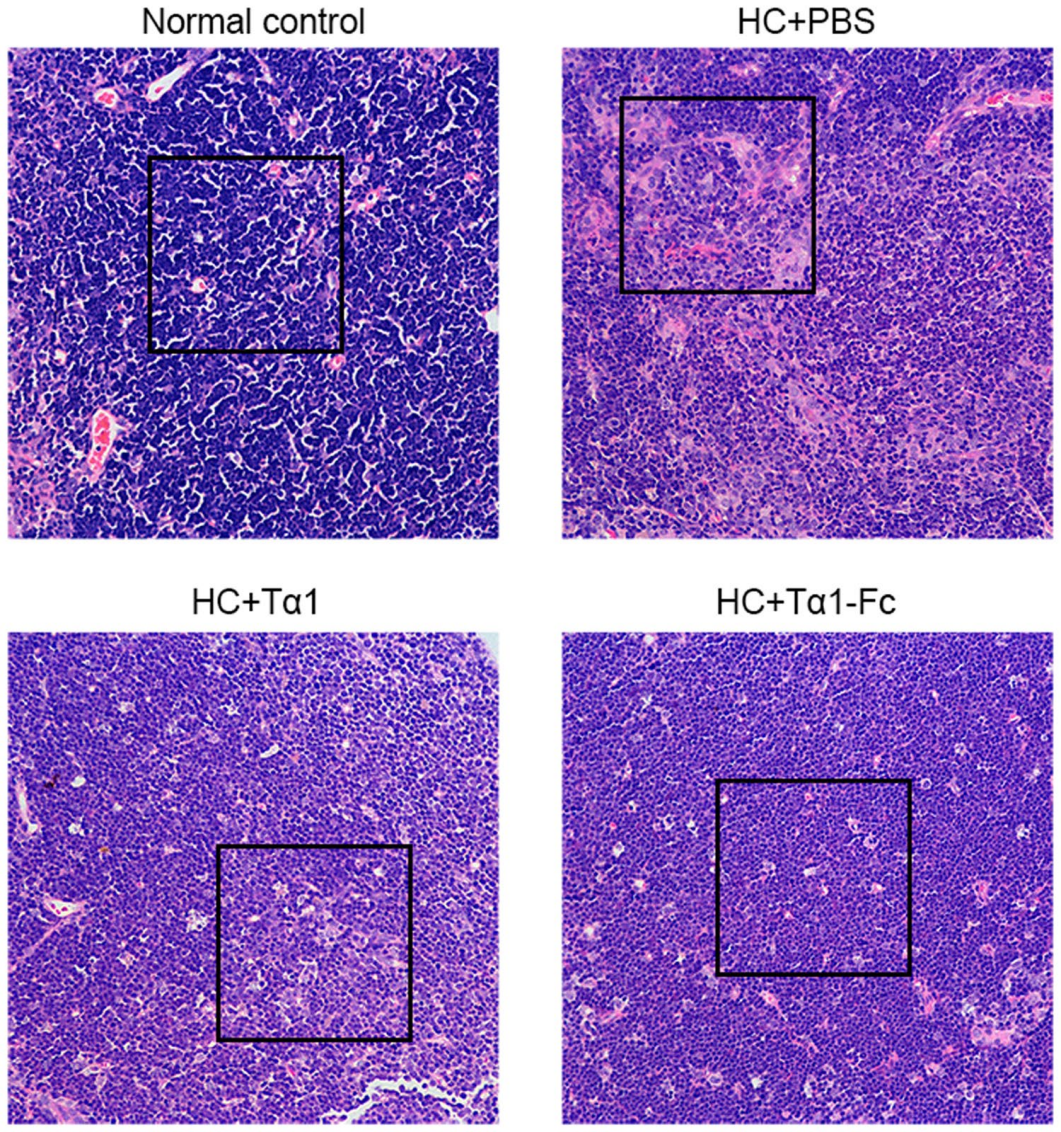
H&E staining of thymus tissues. Parts in black rectangles shows a comparison of thymocytes necrosis between different groups. The nucleus was stained as blue by hematoxylin and the cytoplasm was stained as pink by eosin. Scale bars, 50 μm.

H & E was used to detect whether the cells are damaged; normal thymocytes appeared to be dark blue, and the damaged one is light pink. The pink area accounts for a large part, which reveals that the number and the condition of thymocytes in immunocompromised mice treated by HC declined significantly (Fig. 4). Color from light pink to deep purple are groups treated with PBS, Tα1, and Tα1-Fc, as well as the normal control group; this finding shows that Tα1-Fc has a stronger function in repairing the damaged thymus than among Tα1.

### Tα1-Fc exhibited a better anti-tumor activity than Tα1 on 4T1 mouse mammary tumor xenografts

**Figure 5:**
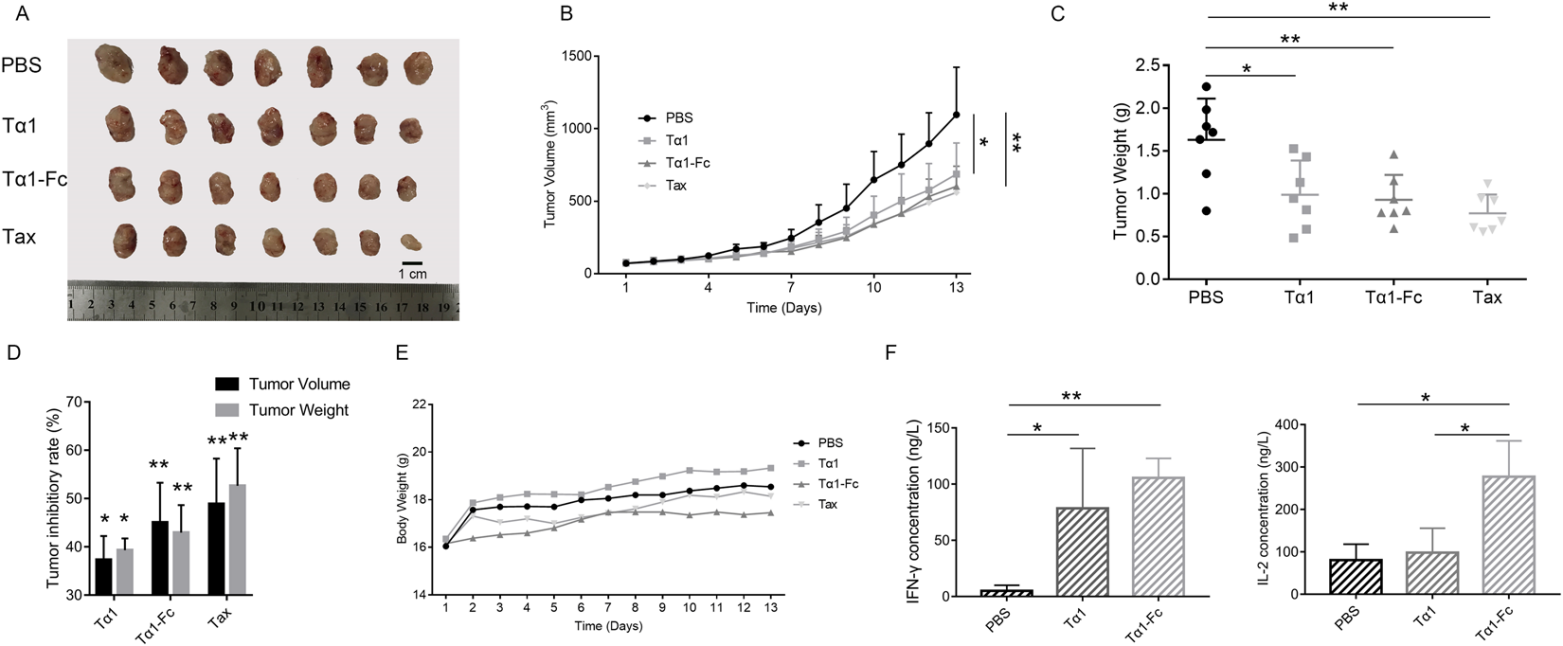
Tα1-Fc exerted a stronger tumor growth inhibitory compared to Tα1 in 4T1 model (n = 7). (**A**) 4T1 tumor entity photo. (**B**) The tumor volume. (**C**) The tumor weights. (**D**) The tumor inhibitory rate. The difference was analyzed compared with PBS group. **(E)** The body weights of BALB/c. (**F**) The concentration of peripheral blood IFN-γ and IL-2.

4T1 tumor model in BALB/c mice is an animal model for stage IV human breast cancer that closely mimics human breast cancer. The growth of 4T1 tumor was relatively slow in the first 6 days after the first drug injection. As time progressed, a difference between independent groups became larger. On day 13, mice were treated with cervical dislocation to obtain the tumor entity (Fig. 5A) and peripheral blood. The average tumor volume of PBS group reached 1,097.19 ± 327.51 mm^3^, whereas that of Tα1, Tα1-Fc, and Tax is 687.61 ± 199.08, 602.84 ± 138.99, and 560.74 ± 112.49 mm^3^, respectively (Fig. 5B). The tumor weight trend was consistent with the volume growth trend, which obtained PBS > Tα1 > Tα1-Fc > Tax at 1.63 ± 0.48, 0.99 ± 0.40, 0.93 ± 0.29, and 0.77 ± 0.22 g, respectively (Fig. 5C). The inhibitory activity of Tα1 and Tα1-Fc was 37.33% and 45.06% on tumor volume and 39.31% and 42.96% on tumor weight (Fig. 5D, Table 3), respectively. In comparison with PBS, Tα1 and Tα1-Fc inhibited the tumor growth strongly with no significant side effects (p = 0.0171, p = 0.0032). However, mice treated with Tax appeared to be thin and have lost weight. Weight of mice treated with Tax was 17.94 ± 0.78 g, whereas that of the PBS group was 18.82 ± 1.49 g (Fig. 5E). In this finding, Tα1-Fc and Tax showed a significant difference compared with PBS. In 4T1 xenograft tumor models, the s.c. administration of Tα1-Fc showed a stronger inhibitory activity than Tα1 on tumor volume and tumor weight.

**Table 3:** The tumor inhibitory rate of tumor volume and tumor weight in 4T1 model (n = 7).

Inhibition Rate (%)	Tα1	Tα1-Fc	Tax
Tumor Volume	37.33 (P = 0.0171)	45.06 (P = 0.0032)	48.89 (P = 0.0015)
Tumor Weight	39.31 (P = 0.0189)	42.96 (P = 0.0063)	52.66 (P = 0.0010)

The difference was analyzed compared with PBS group.

Cytokine secretions, e.g. IFN-γ and IL-2, can be regulated by Tα1. IFN-γ has the capability to modulate the immune response against a variety of antigens, whereas IL-2, as an anti-inflammatory cytokine, can boost the host immunity against cancer^23,24^. ELISA was performed to detect concentrations of IFN-γ and IL-2 in peripheral blood. Cytokine concentration was increased in either Tα1 group or Tα1-Fc group, especially the IFN-γ concentration of Tα1-Fc (p = 0.00003). Also, the IL-2 concentration of Tα1-Fc had a significant difference with that of Tα1 (p = 0.0389) (Fig. 5F). In all, Tα1-Fc took advantage of Tα1 in the secretion of cytokines IFN-γ and IL-2.

**Figure 6:**
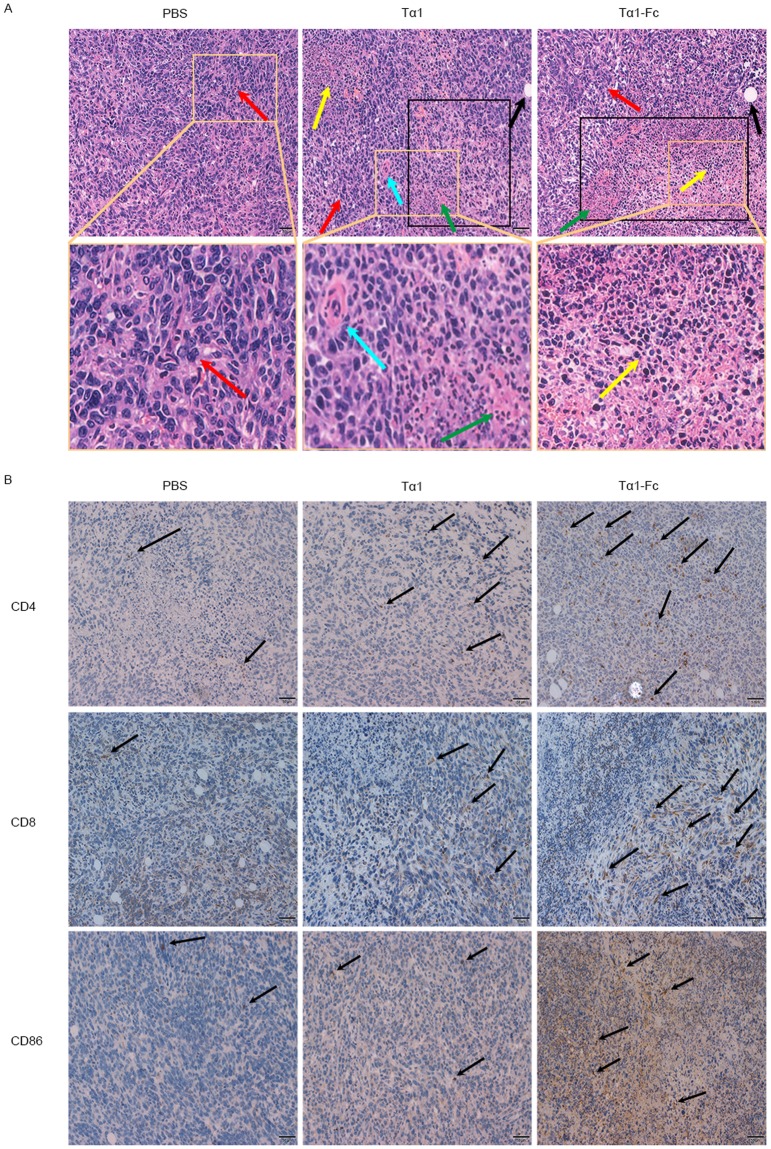
The H&E staining and IHC of 4T1 tumor tissues. (**A**) H&E staining. Vacuoles, black arrow; giant multinucleated cells, blue arrow; pathologic mitotic phase, red arrow; inflammatory cells, yellow arrow; necrosis, green arrow and black rectangle. Scale bars, 50 μm. (**B**) The influence of drugs on expression of CD4, CD8 and CD86. Scale bars, 1 mm.

The H&E staining slices of 4T1 tumor are shown in Fig. 6A. The vast majority of 4T1 cells in the PBS group were in a good proliferative state with a nuclear structure. The partially pathologic mitotic phase is marked by yellow arrow. Vacuoles and giant multinucleated cells can be seen in the slice of the Tα1 group with local tissue necrosis. By contrast, the Tα1-Fc group showed a large-scale organization of necrosis with cytoplasmic eosinophilic, cell atrophy, and inflammatory cell (eosinophils, neutrophils, and mononuclear cells) infiltrations in the tumor tissue.

CD4 is a co-receptor for Ag recognition and presentation, whereas CD8+ T cells can lysis tumor cells. In this study, CD4, CD8, and CD86 were detected by IHC. Results are shown in Fig. 6B. In 4T1 tumor models, mice treated with Tα1-Fc, compared with Tα1, showed an increased expression of CD86 and promoted CD4+ T lymphocytes and CD8+ T lymphocytes infiltrating tumor tissues.

### Tα1-Fc displayed a stronger tumor growth inhibitory on melanoma compared with Tα1

**Figure 7:**
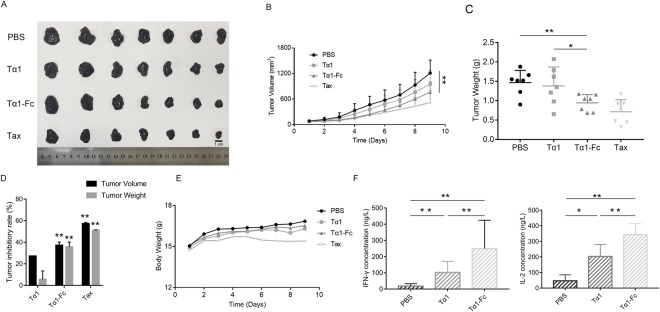
The antitumor activity on inhibiting tumor growth and secretion of cytokines on melanoma (n = 7). (**A**) Melanoma tumor entity photo. (**B**) The tumor volume. (**C**) The tumor weights. (**D**) The tumor inhibitory rate. The difference was analyzed compared with PBS group. (**E**) The body weights of C57BL/6. (**F**) The concentration of peripheral blood IFN-γ and IL-2.

The melanoma growth is explosive and it took only nine days from the first administration to be executed. A solid tumor photo is shown in Fig. 7A. On day 9, the average tumor volume of PBS was about 1,200 mm^3^, whereas those of Tα1, Tα1-Fc, and Tax are 881.71 ± 305.2, 761.02 ± 239.85, and 518.21 ± 280.74 mm^3^ (Fig. 7B), respectively. Administration of Tα1-Fc and Tax significantly reduced the tumor volume with P value of 0.009 and 0.008, respectively. Also, the tumor weight of Tα1-Fc (0.9420 ± 0.2152) showed a significant decline than of Tα1 (1.3810 ± 0.4859, p = 0.0494) (Fig. 7C). The tumor inhibitory rate is 27.25%, 37.21%, and 57.24% of Tα1, Tα1-Fc, and Tax, respectively (Fig. 7D, Table 4). The average mice weight of Tax declined about 0.33 g from the fifth day to the ninth day, whereas other groups basically remained stable (Fig. 7E). Other side effects, such as poor appetite, were observed just like on the 4T1 models treated by Tax. Tα1-Fc had no effect on mouse weight. Thus, Tα1-Fc exerted a better anti-tumor activity compared with Tα1 on melanoma.

**Table 4:** The tumor inhibitory rate of tumor volume and tumor weight in melanoma (n = 7).

Inhibition Rate (%)	Tα1	Tα1-Fc	Tax
Tumor Volume	27.25 (P = 0.0654)	37.21 (P = 0.0096)	57.24 (P = 0.0008)
Tumor Weight	5.71 (P = 0.7091)	35.69 (P = 0.0035)	51.24 (P = 0.0007)

The difference was analyzed compared with PBS group.

On melanoma models, IFN-γ and IL-2 were upregulated by either Tα1 or Tα1-Fc in which the concentration of the two cytokines of the Tα1-Fc group showed a significant difference compared with that of the PBS group (p = 0.0016, p = 0.0032) (Fig. 7F). Findings show that the concentration of IFN-γ and IL-2 stimulated by Tα1-Fc increased by several times compared with the Tα1 group. These results suggest that Tα1-Fc may inhibit tumor progression by the secretion of cytokines IFN-γ and IL-2.

**Figure 8:**
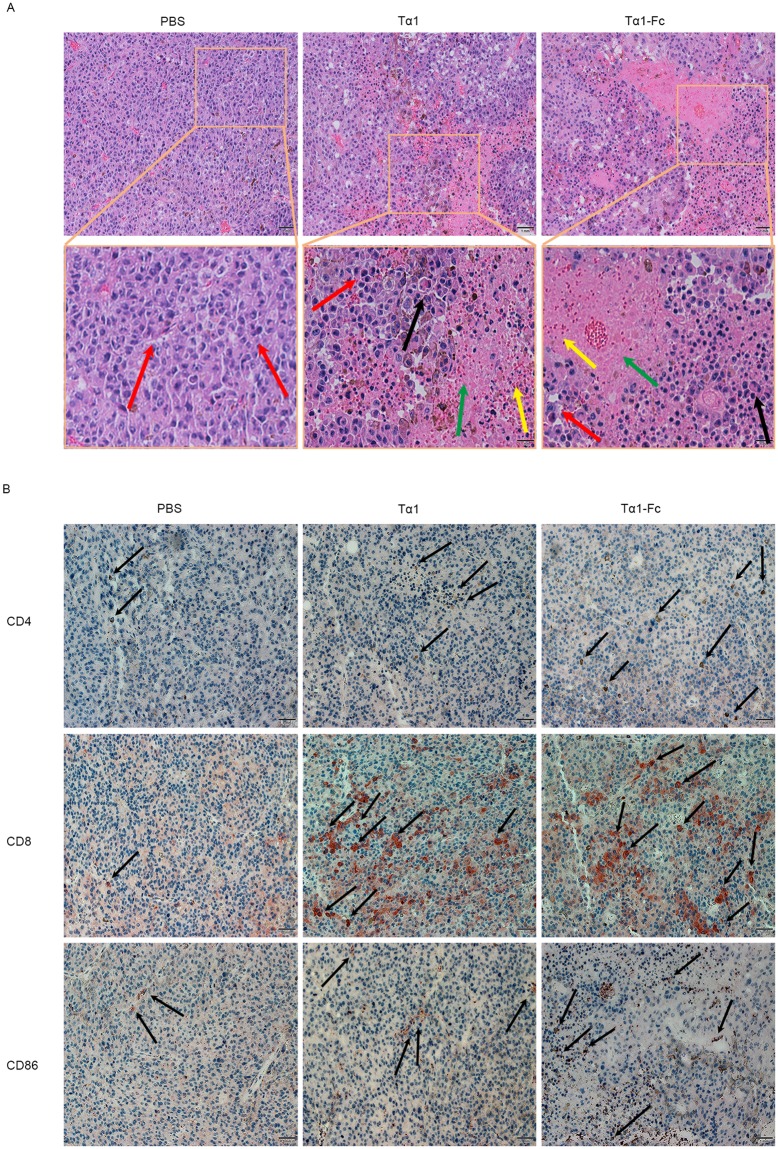
The H&E staining and IHC of melanoma tissues. (**A**) H&E staining. Vacuoles, black arrow; giant multinucleated cells, blue arrow; pathologic mitotic phase, red arrow; inflammatory cells, yellow arrow; necrosis, green arrow and black rectangle. Scale bars, 50 μm. (**B**) The influence of drugs on expression of CD4, CD8 and CD86. Scale bars, 1 mm.

Necrosis in B16F10 tumor tissues is shown in Fig. 8A. A part of B16F10 cells were in the mitotic phase in the PBS group. Local tumor tissue necrosis appeared with some inflammatory cells infiltrating in the Tα1 group, whereas that of the Tα1-Fc group was larger with more shrinking cells.

On the aspect of CD molecular expression, increased CD4 and CD86 were detected on the melanoma models treated with Tα1 and Tα1-Fc (Fig. 8B). Tα1-Fc showed a stronger effect on CD4+ T lymphocyte infiltration than that of Tα1. More CD86 were observed in the necrosis treated with Tα1-Fc. The background color interfered with the detection of CD8 when DAB staining was used in melanoma; hence, ACE was chosen for staining CD8. Tα1-Fc promoted CD8+ T lymphocytes, which infiltrate tumor tissues; this finding is comparable with that of Tα1 group. In summary, Tα1-Fc inhibited the tumor growth on melanoma with an increased expression of IFN-γ, IL-2, and CD86 and of tumor-infiltrating CD4+ T and CD8+ T cells.

## Discussion

Tumors often occur in immunosuppressed individuals with declined DC functions^25^. Cellular immune response efficiency depends on Ag capture, processing, delivery to lymph nodes, and presentation to effector cells of the adaptive immune system. Tα1, as an immunomodulator, has dual effects on DC functions in sensing infection and tissue stress through stimulating TLR agonists^26^ and on tumor cells by upregulating major histocompatibility complex class-I Ag expression in normal and transformed cells that resulted in an increased Ag presentation. In addition, the production of cytokines intervenes tumor progression and development.

Tα1 was recently proved to bind with human serum albumin (HSA)^27^. HSA is an important protein in serum and serves as a carrier for many drugs and peptides. The binding of Tα1 to HSA might help to diffuse Tα1 along the blood circulation. These results shed the light on pharmacokinetic properties of Tα1. To find out whether the Tα1-Fc maintain these pharmacokinetic properties or not, we also plan to study the pharmacokinetic aspects of Tα1-Fc, and its mechanism in the next step in the future.

Tα1 was also recently proven to interact with hyaluronic acid (HA) by its C-terminal sequence LKEKK^28^. Tα1 shares the similar sequences with CD44 and RHAMM which both can bind with HA. HA, a kind of glycosaminoglycan, plays an important role in a variety of diseases, and developmental and physiological processes. Tα1 was proven to inhibit the HA-CD44 or HA-RHAMM interactions and then suppress tumor progression. Based on these findings, further research on the interaction of Tα1-Fc with receptors or extracellular matrix components like HA need to be explored in the future for a better understanding of the immune and antitumor mechanisms.

Tα1 is a natural circulating hormone peptide capable of influencing many components of the inflammatory/autoimmune cascade at a time. Considering the short half-life of Tα1, we constructed a fusion protein of Tα1 and IgG Fc fragment. Most antibodies approved by FDA are composed of IgG1, in that IgG1 shows a stronger affinity to FcγR than IgG4 which can induce ADCC to enhance the antitumor activity. Among these antibodies, Portrazza, Perjeta and Erbitux are the most famous used in the treatment in HNSCC, breast cancer and CRC, respectively. However, Tα1 is a non-target protein. In order to reduce the ADCC and CDC caused by the combination of Fc and FcγR, IgG4 was chosen. The fusion protein glucagon-like peptide-1 (GLP-1) is one of many success stories by introducing IgG4-Fc fragment^29^. For now, there are some other IgG4-Fc fusion proteins been put on the market, such as Mylotarg (calicheamicin, IgG4), Opdivo (PD-1 mAb, IgG4), Keytruda (PD-1 mAb, IgG4). In summary, the longer serum half-life and the lower risk of adverse effects reinforce IgG4-antibody development.

The half-life was determined on normal Wistar rat via tail vein injection. The plasma concentration of the synthetic peptide Tα1 was reduced by half at approximately 2 h, whereas Tα1-Fc at about 25 h. On the one hand, the theoretical MW value of recombinant protein increases to 46 kDa, with TrxA 17 kDa and Tα1-Fc 29 kDa. On the other hand, the introduction of the Fc region allows the binding with FcRn that prevents IgG dissociation from FcRn and release into the bloodstream, rather than directing IgG into a degradation pathway^16^; this finding results in an increased MW larger than the glomerular filtration value of about 69 kDa^18^. Improved pharmacokinetic properties contribute efforts for clinical use of Tα1^30^. However, serum Tα1 levels varied considerably among different individuals and different diseases^31–33^, and it is difficult to discriminate endogenous protein from exogenous protein by ELISA. Given individual differences in mice, the circulating levels of endogenous Tα1 in relation to the exogenously administered Tα1-Fc will be determined by using radiolabeled proteins in the future.

In addition, Tα1 restores NK activity and reconstructs cell immunity in immunosuppressed mice^12^. In this study, we evaluated the immune function in immunocompromised ICR mice. One week after HC withdrawal, the thymus and the spleen did not regain their normal size. Thymus is the major organ for producing T lymphocytes and numerous cytokines and thymic hormones. Our findings showed that immunosuppressed mice treated with Tα1 and Tα1-Fc improved varying degrees, specifically in the thymus index and the spleen index. Tα1-Fc can also regulate the immune system by stimulating cytokine production, such as IFN-γ and IL-2. Certainly, the recombinant protein exhibited a better activity on reconstructing the immune system compared with synthetic peptide Tα1.

Tα1 has been proved effective in several cancers, such as lung cancer^34^, colon cancer^35^, melanoma^10^, and breast cancer^36^. In this study, we investigated the *in vivo* antitumor activity of Tα1-Fc on B16F10 and 4T1 tumor models. The tumor volume trend chart and tumor weight chart all demonstrated that Tα1-Fc can inhibit tumor growth stronger than Tα1. The CD4 and CD8 co-receptors are predominantly expressed on the surface of T helper cells (Th) and cytotoxic lymphocytes (CTL), respectively. Immune response requires CD4 for Ag recognition in cooperation with CD8 for tumor elimination. However, CD8 T cells with low avidity for tumor Ag were inefficient in tumor invasion^37^. Studies have proved that CD4+ T cells exert antitumor activities by activating and recruiting macrophages and eosinophils, which produce tumor-destroying free radicals and induce the secondary expansion and accumulation of CD8+ T cells by co-expressing IL-21 and IFN-γ^38,39^. In addition, Th1 cells in the early stages were changed to Treg and Th17 cells in the late stages of the breast cancer development as instructed by CD4+ T cells^40^. Tumor-infiltrating CD8+ T lymphocytes play a dominant role in killing tumor cells by secreting cytokines or chemokines, which have antitumor effects. Further studies demonstrated that tumor-infiltrating CD8+ T cells take part in assessing disease prognosis and clinical progression^41^. Likewise, CD86 is an activation receptor for NK cell cytotoxicity against tumor cells^42^ and CD86 on non-bone marrow-derived cells prime CTL in DNA vaccine trials^43^. The slices of 4T1 and melanoma models exhibited an increased number of CD4, CD8, and CD86 in the Tα1-Fc group compared with that in the Tα1 group.

*In vivo* findings detailed in this paper reinforce the validity of this recombinant protein as an immune-enhancing agent and an antitumor compound by stimulating the secretion of cytokines and by upregulating CD86 to a modest number. These findings strongly encourage the further exploitation of Tα1-Fc in clinical use for cancer therapy.

## Conclusion

In general, the recombinant protein we produced has a stronger activity in stimulating cytokine secretion and repairing damaged immunity system. Our findings also demonstrate a better tumor inhibitory on melanoma and 4T1 mouse mammary tumor xenograft. These findings reinforce the potential use of Tα1-Fc as a promising antitumor compound against different tumors.

## Materials and Methods

### Materials

4T1 murine breast cancer cell line and B16F10 melanoma cell line were purchased from American Type Cell Culture (ATCC, Shanghai, China). Hydrocortisone was purchased from Tianjin Jinyao Pharmaceutical Co., Ltd. Paclitaxel (Taxol) was purchased from Beijing Concord Pharmaceutical Co., Ltd. Tα1 were synthesized by China Peptides Company Limited (Shanghai, China). ICR, BALB/c, C57BL/6 mice, and Wistar rats were purchased from the Comparative Medicine Center of Yangzhou University, China. Anti-CD4 antibody, anti-CD8 antibody, anti-CD86 antibody, mouse IFN-γ enzyme-linked immunosorbent assay (ELISA) kit, and mouse IL-2 ELISA kit were purchased from Shanghai Bioye Biological Technology Co., Ltd. Rat Tα1 ELISA kits were purchased from Shanghai MLBIO Biotechnology Co., Ltd. All the animal experiments complied with the rules and regulations of Contract 2016(su)-0010 with the approval of Jiangsu Provincial Experimental Animal Management Committee.

## Methods

### Expression of recombinant protein Tα1-Fc

Plasmid pET32a containing the selected gene Tα1-Fc was transformed into BL21(DE3) cells and incubated in LB medium (30 mL) with ampicillin (final concentration 100 µg/mL, the same below) 200 rpm for 12–14 h at 37 °C. The solution was placed on LB-agar plate for 12–14 h. A single colony was picked and reincubated with the same condition later. The seed liquid was inoculated in the medium in a proportion of 1% of the bacteria solution. Afterwards, the fusion protein was induced by different concentrations of IPTG or lactose when the OD_600_ of the transformed incubated bacteria reached 0.6–0.8. The solution was centrifuged at 8,000 rpm and resuspended in bacterial suspension buffer. Broken bacteria were obtained by using ultrasonic crusher.

### Nickel ion affinity chromatography

Most Fc antibodies can be purified via standard protein A or G chromatography. However, the content and production of Tα1-Fc obtained by Protein A or G are low. Owing to the His6-tag in the expressed product, we can purify Tα1-Fc by using Ni2+ affinity chromatography^21,44^. The sample solution of the two columns was added into the Ni2+ column for combining with Ni2+ in a fixed rate; afterwards, the imidazole solution of the gradient concentration of about ten columns was added successively to compete with the His6 combination. Elution was analyzed by using SDS-PAGE. To obtain lyophilized protein powder, the elution was desalted with 0.1 mol/L ammonium bicarbonate by initially using Sephadex G-25, and the elution was then lyophilized later.

### Western blotting

Acrylamide concentrations of concentrating and separating gels for SDS-PAGE were 5% and 15%, respectively. A total of 20 µL sample and 5 µL 5 × Loading Buffer were mixed and then denatured in boiling water for 5 min. The mixture was added into gel wells with 10 µL. The gels were run at 80 V and then 120 V when the bands reached the separation of gels and bottom of gels. All gels were imaged using Image Master VDS. After SDS-PAGE, gels were transferred to nitrocellulose membranes and run at 160 mA for 1.5 h at 4 °C, then blocked in 5% skim milk for an hour. Primary anti-huaman-IgG4 antibodies and secondary antibodies were successively added for 2 and 1.5 h incubations, respectively. Detection was performed by using ECL system.

### Mass spectrometry

Protein identification was performed with a UltraflexTOF/TOF mass spectrometer (Bruker Daltonics Co., Ltd). The instrument was operated in linear positive mode. Protein eluted from Ni2+ affinity column was identified as pure by SDS-PAGE. Sinapinic acid was prepared in a saturated solution of TA50 (0.1% TFA) and added to the sample droplet in a 1:1 ratio (v:v). The mass spectrometric data are technical triplicates by three times sample-injecting.

### *In vivo* determination of serum half-life

Wistar rats were treated with a single intravenous injection at a dose of 0.057128 µmol/kg drug/rat. Peripheral blood was subsequently collected with sodium citrate for anticoagulation after 10 min, 30 min, 1 h, 1.5 h, 2 h, 3 h, 6 h, and 12 h or after 15 min, 1 h, 5 h, 13 h, 30 h, 48 h, 72 h, and 100 h and then centrifuged immediately. Plasma Tα1 and Tα1-Fc concentrations were determined by using a Rat Tα1 ELISA kit. SARS was used for data processing.

### Immunocompromised mice models

Mice were treated with 50 mg/kg HC via subcutaneous injection every day for a week and then divided into three groups (PBS, Tα1, and Tα1-Fc 0.081532 µmol/kg) randomly when the rats obtained body malaise and weight loss plus a blank control group without any treatment. Mice were sacrificed by cervical dislocation to obtain the thymus and spleen, which were detected by hematoxylin and eosin (H&E) after 7 days. Peripheral blood was allowed to stand for at least 30 min, centrifuged at 4,000 rpm for 10 min (the same below), and then determined by using a mouse IFN-γ ELISA kit and a mouse IL-2 ELISA kit.

### Tumor modeling

4T1 tumor cells and B16F10 melanoma cells were injected into syngeneic BALB/c and C57BL/6 mice, respectively, at 1 × 10^5^/mL concentration. These mice were divided into four groups randomly (PBS, Tα1, Tα1-Fc 0.081532 µmol/kg, and Tax 0.011711 µmol/kg) with seven mice each group when the tumor volume reached 80 mm^3^. The mice were treated with PBS, Tα1, Tα1-Fc every day, or Tax every 2 days. Tumor volume and body weight were measured every day. When the PBS group’s average tumor volume reached 1,000 mm^3^, the solid tumors were obtained and stored in 4% paraformaldehyde.

### ELISA

ELISA is widely used for quantitating antibodies(Ab) or antigens(Ag) by utilizing an enzyme-linked antibody binding to a surface-attached Ag^45^. Blank wells and standard wells with five gradients and sample wells were used; each well was added 50 µL sample. The plate was incubated at 37 °C (the same below) for 30 min then incubated with 50 µL of HRP-labeled goat anti-mice antibody, except the blank well after washing the plate. TMB-A and TMB-B were added for coloring following the end solution after 10 min. The absorbance of each well was read on a spectrophotometer using 450 nm as the primary wavelength.

### Histochemistry and immunohistochemistry

Cell pathological changes, such as tissue necrosis, were detected by using histochemistry (H&E) staining^46^. Immunohistochemical (IHC) staining of CD molecular was used to evaluate tumor-infiltrating T cells in the tissues. Briefly, the fixed tissue was subjected to dehydrated, transparent, process-dip wax, embedding, sectioning, sticky sheet, and bake sheet to obtain tumor tissue sections with about 4 µm thickness. Sections were incubated with H_2_O_2_ (3%) for 20 min, then with the primary antibodies (rat anti-mouse CD4 or CD8 or CD86 monoclonal antibody) for 12 h, and the secondary antibody (HRP-tagged rabbit anti-rat IgG) for 1 h successively. Next, tissue slices were treated with 3,3-diaminobenzidine (DAB) or 3-amino-9-ethylcarbazole (ACE) and re-stained by hematoxylin. At last, slices were covered by coverslips and stored at room temperature.

### Statistical analysis

All data were presented as mean ± SD. The statistical significance of all results was evaluated by using one-way ANOVA followed by post hoc Tukey HSD test using R Software Version 3.3.1.; *p < 0.05; **p < 0.01.

## Electronic supplementary material

Figure 3 -dataset

Figure 4-dataset

Figure 5–6-dataset

Figure 7–8-dataset

## Data Availability

The datasets generated and analyzed during the current study are available from the corresponding author on reasonable request.
